# Evaluation of Caffeine Consumption among Pregnant Women from Southern Poland

**DOI:** 10.3390/ijerph15112373

**Published:** 2018-10-26

**Authors:** Ewa Błaszczyk-Bębenek, Beata Piórecka, Monika Kopytko, Zuzanna Chadzińska, Paweł Jagielski, Małgorzata Schlegel-Zawadzka

**Affiliations:** Human Nutrition Department, Institute of Public Health, Faculty of Health Sciences, Jagiellonian University Medical College, Grzegórzecka 20, 31-531 Krakow, Poland; beata.piorecka@uj.edu.pl (B.P.); monikak1019@gmail.com (M.K.); zchadzinska@poczta.fm (Z.C.); pawel.jagielski@gmail.com (P.J.); m.schlegelzawadzka@gmail.com (M.S.-Z.)

**Keywords:** caffeine, pregnancy, nutritional habits

## Abstract

Caffeine is the most widely consumed stimulant worldwide, including by pregnant women. Products containing caffeine should be limited in accordance with the recommendations for pregnancy. The purpose of this study was to evaluate consumption of caffeinated products and daily caffeine intake from food by pregnant women. The study was conducted on a group of healthy pregnant women: attendees of antenatal classes (*n* = 70) and patients of an outpatient gynecological clinic (*n* = 70) from Krakow (Southern Poland). A questionnaire about the frequency of consumption of selected foods and drinks containing caffeine was used. The average caffeine content in food products obtained from other Polish studies was used to estimate average daily caffeine intake in our study group. Mean daily caffeine intake was 49.60 ± 59.15 mg/day and the maximum was 498.0 mg/day. The main sources of caffeine were as follows: black tea (bags, leaf), instant coffee and ground coffee. No statistically significant differences in caffeine intake between the groups were found. A weak negative correlation (rs) = −0.28 (*p* = 0.0208) between month of pregnancy and caffeine intake was observed among attendees of antenatal classes. Mean daily caffeine intake did not exceed the maximum recommended dose in our study group.

## 1. Introduction

Adequate nutrition in pregnancy remains one of the key factors for normal fetal development. Nutritional recommendations for pregnant women encompass both quantitative norms and qualitative compliance, including meal planning [1,2,3]. Limited consumption of strong tea and coffee has been recommended in pregnancy. Caffeine found in these two products is one of the most common stimulants in the world and its stimulating properties affect the nervous and cardiovascular systems [4,5,6,7]. Caffeine is found not only in drinks based on natural infusions, such as coffee or tea, but also in cocoa and cocoa-based products, as well as medicines. Furthermore, cola-like products and energy drinks, which contain synthetic caffeine, are also available on the market [4]. Caffeine passes the placental barrier freely and is found in the amniotic fluid as well as maternal milk, which might have negative consequences for the developing fetus and the newborn [8]. The metabolism of caffeine depends on various factors, including the stage of pregnancy. As the pregnancy progresses, caffeine half-life extends to 11–18 h, especially in the third trimester [9,10]. In pregnancy, caffeine consumption over the recommended safe levels may be associated with low birthweight [10], or neonatal sleep apnea [9]. Also, the risks of premature labor and miscarriage are elevated in women who consume high amounts of caffeine [10]. Excessive use of products with a high content of caffeine, which reduces iron bioavailability, may also be associated with maternal anemia in pregnancy [10,11]. Various authors investigated the possible relationship between products containing caffeine and female fertility and found that chances for conception are lower in women who consume over 300 mg/day, especially among smokers. Embryotoxicity of caffeine consumption at the dose of >300 mg/day during pregnancy has been confirmed by numerous studies [10,11,12]. In 2015, the European Food Safety Authority (EFSA, Parma, Italy) lowered their recommendations regarding the safe amount of caffeine content in the diet of pregnant women from 300 mg to 200 mg/day [13]. According to EFSA, regular daily caffeine intake of 200 mg during pregnancy poses no threat to fetal development. At the same time, a single safe dose of caffeine intake for pregnant women has not been determined. Poland lacks guidelines in that regard; however, the need to reduce caffeine consumption due to potential risk for the fetus has long been emphasized [10]. The aim of this study was to present and evaluate the consumption of foods and drinks containing caffeine by pregnant women from Krakow, and to assess daily fluid and caffeine intake in the study group.

## 2. Materials and Methods

The study was conducted in two groups of women in normal, healthy pregnancy (single, unthreatened gestation). The first group included attendees of antenatal classes (*n* = 70) located at three Krakow hospitals, and the second group consisted of patients of an ob-gyn outpatient clinic (*n* = 70), also from Krakow. The former group was evaluated in September 2014 and the latter between June–July 2015, using the same tool. Overall, a total of 140 women (aged 20–41 years) in the second and third trimesters of pregnancy were included in the study.

The study was conducted in accordance with the Declaration of Helsinki for medical research [14]. Written informed consent was obtained from all participants. All women were notified about the possibility of withdrawing from the study.

An original questionnaire, designed for a previous study [15] on the basis of the Questionnaire of Eating Behavior (QEB) [16] and dairy products frequency questionnaire (ADOS-Ca) [17], was used to assess the amount and frequency of consumption of selected caffeinated products (drinks and chocolate). Also, we attempted to evaluate average daily caffeine intake.

Frequency of caffeine consumption, constituting the qualitative measure of caffeine intake, was evaluated using one out of eight possible answers: 3 times/day, 2 times/day, 1 time/day, 5–6 times/week, 3–4 times/week, 1–2 times/week, less than 1 time/week, never. The quantitative measure of caffeine intake was evaluated using the portion of the caffeinated product. Daily portion sizes were determined as follows: 75 mL, 150 mL, and >150 mL for coffee; 125 mL, 250 mL, and >250 mL for tea, cola-like drinks and energy drinks; and, 50 g, 100 g and >100 g for chocolate. The following products were included: ground coffee, instant coffee, black tea (bags), black tea (leaf), green tea (bags), green tea (leaf), cocoa, cola-like drinks, energy drinks, and, bitter and milk chocolate. Mean caffeine intake from selected products obtained by other Polish authors was used to assess mean caffeine intake in the daily diets of our study population [18,19,20]. Neither the brewing times for tea and coffee nor the amount of the product used during a brew cycle were taken into account in our study (Table 1).

Daily intake of all fluids in the diet was obtained based on caffeinated and non-caffeinated beverages consumed in pregnant women’s diets.

The questionnaire also included socioeconomic data (place of residence, marital status, financial status, professional status, education) and nutrition status (weight, height) of the study population. Pre-pregnancy body mass index (BMI) was calculated for each woman and used to evaluate their nutrition status, according to the WHO criteria. 

The participants were subdivided into two groups: attendees of antenatal classes (group 1) and patients of an ob-gyn outpatient clinic (group 2). Chi square or Mann-Whitney U tests and Spearman’s rank were used to determine intergroup and intervariable dependencies. The *p*-value of α = 0.05 was considered as statistically significant. STATISTICA 10 PL (StatSoft, Poland) was used for statistical analysis.

## 3. Results

A total of 140 women (mean age 29.81 ± 4.2 years) were included in the study. Mean age in group 1 (30.36 ± 3.26 years) was significantly higher (*p* = 0.0281) as compared to group 2 (29.26 ± 4.92 years). Detailed social and demographic characteristics of the study population are presented in Table 2. 

Data concerning pregnancy and health behaviors during the course of the pregnancy are presented in Table 3. All respondents declared total alcohol abstinence during pregnancy.

Mean total caffeine consumption in the study population was 49.60 ± 59.15 mg/day. Minimum and maximum caffeine intakes in the diet were 0.00 mg/day and 498.0 mg/day, respectively. The median daily caffeine intake in the study group was 33.49 mg, while 25% of respondents exceeded 65.11 mg. Only two women consumed over 200 mg caffeine within a day, the permissible limit. No statistically significant differences in total caffeine intake from selected products were found between the groups (Antenatal classes 44.38 ± 38.26 mg/day vs. Gynecological clinic 54.82 ± 74.35 mg/day; *p* = 0.9837). 

**Figure 1:**
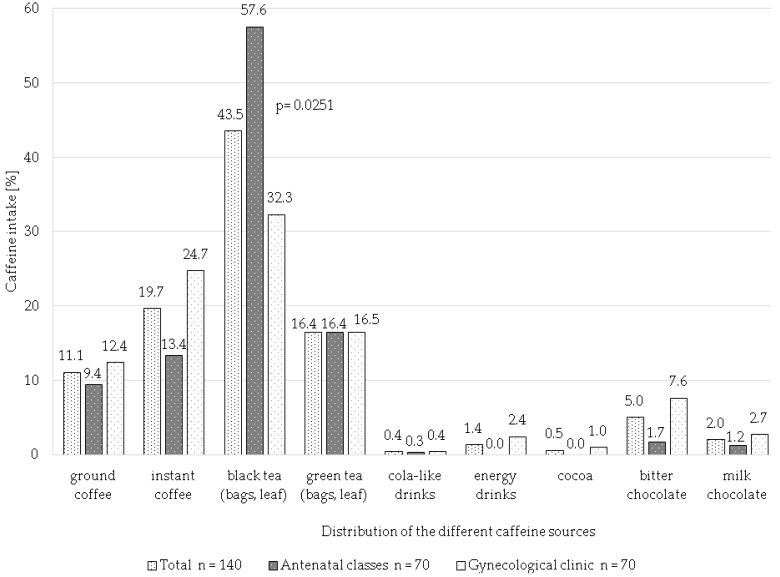
Daily caffeine intake versus source of caffeine, in total and by group (%).

Black tea (bags and leaf) proved to be the main source of caffeine (21.61 mg/day), followed by instant coffee (9.75 mg/day), and ground coffee (5.50 mg/day). No statistically significant differences between the groups were found except for black tea bags and leaf (Antenatal classes 25.55 ± 31.33 mg/day vs. Gynecological clinic 17.67 ± 27.32 mg/day; *p* = 0.0251). The structure of distribution of daily caffeine intake (%), from various sources, in total and by group, is presented in Figure 1.

A weak negative correlation (rs) = −0.28 (*p* = 0.0208) between the month of pregnancy and caffeine intake was observed among attendees of antenatal classes. Gradual reduction of caffeinated products was noted with the advancement of pregnancy. However, this may be associated with a significant difference in the month of pregnancy, depending on the place of examination.

Predictably, attendees of antenatal classes consumed black tea (bags) significantly more frequently (*p* = 0.0430) as compared to patients of a gynecological clinic (Table 4).

No statistically significant differences were found with regard to the choice of daily amount (single portion sizes) of caffeinated product consumption between the groups. In total, black tea (bags) was the most often consumed product. 

Fluid intake from all sources (caffeinated and non-caffeinated beverages) for the total group of women ranged from 600 mL to the maximum of 5600 mL a day (mean: 1758.57 ± 867.81 mL per day). The most common drinks were as follows: still mineral water (627.1 ± 357.6 mL/day), 100 percent fruit and vegetable juices (312.9 ± 267.4 mL/day), herbal teas and brews (225.7 ± 232.4 mL/day), fizzy mineral water (221.4 ± 333.0 mL/day), and black tea (bags) (144.7 ± 204.3 mL/day). A statistically significant difference was found between place of residence and the amount of herbal teas and brews consumption (*p* = 0.0375), which were significantly more often selected by city dwellers. Also, a statistically significant correlation was observed between education and type of drink. Women with secondary education consumed more 100 percent fruit and vegetable juices compared to their peers with higher education (*p* = 0.0131), and also consumed more sweetened fizzy cola-like drinks (*p* = 0.0201), and sweetened still drinks (*p* = 0.0036).

## 4. Discussion

Black tea, followed by instant and ground coffee, was the main sources of caffeine in the diet of pregnant women in our study. In a study by Stefanidou et al., coffee was also the most frequently chosen source of caffeine for pregnant women from Turin (72.3%) [21]. In a study from the United Arab Emirates on caffeine sources in the diet of pregnant women, 61.9% of the women reported coffee to be their drink of choice, followed by tea (34%), and other drinks (4.1%) [22]. Among pregnant women from Warsaw, black tea (58.9%), coffee, including cappuccino (26.8%), chocolate (<3%), green tea and non-alcoholic drinks (5%) constituted the main sources of caffeine in the diet, which is consistent with our findings [23]. Tea consumption between (i.e., not during) meals has been recommended to avoid the risk of anemia as tea may lower iron supply by interfering with iron absorption [11,24]. In light of our findings, it seems reasonable to inform pregnant women about possible sources of caffeine in the diet. In our study, attendees of antenatal classes in Krakow reported reduced consumption of caffeinated products with the progression of pregnancy, which is consistent with the findings of Kobiołka et al., who reported that 70% and 40% of their respondents consumed coffee before and during pregnancy, respectively [25]. Out of 200 pregnant women from the Podkarpackie Region, 70% ceased drinking coffee during pregnancy altogether [26]. 

During pregnancy, caffeine concentration in maternal blood is prolonged due to lower activity of the CYP1A2 isozyme, which in turn results in higher half-life of caffeine. This is especially true of the last trimester of pregnancy and also after delivery [27]. It has been suggested that a higher caffeine intake may be associated with sine causa recurrent miscarriage during the preconception period as compared to normal healthy pregnancy [21]. In the same study from Turin, mean caffeine intake from various sources, evaluated with the use of the same questionnaire as in our study, was 313.5 mg during the preconception period and 150.2 mg (*p* < 0.05) during pregnancy [21]. In our study, mean caffeine consumption was significantly lower (49.6 mg/day). Maximum daily intake of caffeine among pregnant women from Kraków was 498.0 mg, as compared to 547.1 mg in the study from Turin [21]. In a large study from Osaka of 858 pregnant women, median caffeine intake was 258 mg/day [28]. In a group of 509 pregnant women from Warsaw, mean caffeine intake was also higher compared to our study, and was 91 mg/day. The majority of the women from Warsaw consumed ≤100 mg caffeine per day, and only 1.6% of the subjects reported caffeine intake over 300 mg/day [23]. If EFSA guidelines [13] of a maximum daily intake not exceeding 200 mg are taken into account, then in our study only 1.4% of respondents exceed this value. Most (61.7%) pregnant women from the study by Alomar et al. also consumed 200 mg of caffeine per day, and daily intake over 400 mg/day was found in only 6.25% of the cases [22]. Evaluation of caffeine intake during pregnancy is particularly important due to its unrestricted passage across the placental barrier [29]. Various factors affect health decisions made daily by pregnant women, among them education, which is not consistent with our findings or the study by Pieniążek et al. [26]. 

Wikoff et al. conducted a systematic review of potential adverse effects of caffeine consumption in various groups, including pregnant women. Only 3 out of the 58 studies were randomized, and the rest were observational studies using the Food Frequency Questionnaire (FFQ). Caffeine consumption up to 400 mg per day was not negatively associated with the ability to conceive, or fertility, and had a high level of information credibility [30]. Hatch et al., revealed a weak correlation between caffeine consumption of ≥300 mg/day as compared to <100 mg/day and fertility [31]. In a study by Lassi et al., caffeine consumption of >300 mg/day was associated with a higher risk of miscarriage (31%) [32]. A review of 14 studies, which included a total of 130,456 participants, found that each increase in caffeine consumption (by 100 mg) was connected with a higher risk for miscarriage (by 7%). The risk for miscarriage was the highest (1.72 (1.40–2.13) 95% CI) in the group with the highest caffeine consumption (>700 mg/day) [33]. Out of 14 studies on fetal development, 9 reported no effects of caffeine consumption (300 mg/day) during pregnancy on neonatal weight at birth, intrauterine growth restriction (IUGR), placental weight and diameter, neonatal length at birth, or head circumference [30]. Likewise, lack of effects of caffeine consumption (300 mg/day) during pregnancy on pregnancy duration and neonatal condition at birth was reported by the study from Warsaw [23]. The review by Jahanfar and Jaafar also did not confirm the risk for preterm labor or low birth weight in women who consumed on average 182 mg of caffeine per day [34]. According to some authors, fetal exposure to caffeine in the early stages of pregnancy may be associated with excessive weight during childhood [35]. Studies on the effects of caffeine on the course of pregnancy and neonatal condition should be continued and caffeine intake during that time should be restricted [36].

According to the guidelines of the Polish Gynecological Society, pregnant women should drink 3000 mL water/day in the second and third trimester [3]. In our study, only 6.3% of the subjects complied with these recommendations. Kobiołka et al., reported that 28% of their study population followed the guidelines, and water was the drink of choice in 80% of the women, which is consistent with our findings [25]. A Mexican study of 153 pregnant women found their mean daily fluid intake to be 2.62 L, and that the structure of the type of beverage varied with each trimester [37]. According to the study on fluid intake by Guelinckx et al., hot beverages (coffee, tea, and others) were the most frequently consumed drinks, and more often by women than men in Poland. Among the group of Polish respondents, the mean daily intake of hot beverages was 0.73 L/day (0.71–0.75 95% CI) [38].

Our study was not without limitations. The most obvious were a relatively small sample, which was significantly skewed toward better-educated respondents, and no follow-up concerning possible consequences of caffeine consumption in the study group. Additionally, the methodology could be improved to include data based on regular note-taking, and capturing the length of infusion and caffeine intake from other available dietary sources. Hence, further research is necessary.

## 5. Conclusions

Mean caffeine intake in our study population did not exceed the maximum recommended doses for pregnant women. No statistically significant differences were found in total caffeine consumption between the two groups studied. Finally, it seems reasonable to recommend the education of pregnant women about sources of caffeine in their daily diets and the possible risks associated with caffeine consumption.

## Figures and Tables

**Table 1:** Caffeine content of food and beverage sources, used in study.

Source	Average Caffeine Content (mg)	Volume or Weight
ground coffee	60	150 mL
instant coffee	66	150 mL
black tea (bags)	31	250 mL
black tea (leaf)	43	250 mL
green tea (bags)	34	250 mL
green tea (leaf)	41	250 mL
cacao	5	250 mL
cola-like drinks	25	250 mL
energy drinks	80	250 mL
bitter chocolate	67	100 g
milk chocolate	21	100 g

**Table 2:** Social and demographic characteristics of the pregnant women, in total and by group.

Parameter	Total *n* = 140; (%)	Antenatal Classes *n* = 70; (%)	Gynecological Clinic *n* = 70; (%)	*p*-Value
Education				
primary	0.7	0	1.4	0.0184 *
vocational	1.4	0	2.9	
secondary	15	5.7	24.3	
higher	82.9	94.3	71.4	
Financial status				
unsatisfactory	1.4	0	2.9	0.0409 *
satisfactory	15.7	11.4	20.0	
good	62.1	61.4	62.9	
very good	20.8	27.1	14.3	
Marital status				
single	12.9	10	15.7	0.3125 **
married	87.1	90	84.3	
Place of residence				
village	17.4	17.1	27.1	0.1542 **
town	82.86	82.9	72.9	
Accommodation				
independent	16.5	12.9	20	0.2054 **
with family	82.1	84.3	80	
other	1.4	2.8	0	
Professional status				
unemployed	5.7	4.3	10	0.4208 **
regular employment	82.1	90	84.3	
contract	7.1	5.7	5.7	

*n* number of respondents, * Mann-Whitney U test, ** Chi^2^ test.

**Table 3:** Pregnancy and health behaviors of the pregnant women, in total and by group.

Parameter	Total *n* = 140; (%)	Antenatal Classes *n* = 70; (%)	Gynecological Clinic *n* = 70; (%)	*p*-Value
Gravidity				
1	71.4	85.7	58.6	0.0029 *
2	23.6	12.9	32.9	
3	3.6	1.4	5.7	
4	0.7	0	1.4	
5	0.7	0	1.4	
Gestational month				
4	4.3	1.43	5.7	0.0001 *
5	5.0	5.71	7.1	
6	7.1	7.14	7.1	
7	18.6	31.43	5.7	
8	31.4	42.86	20.0	
9	33.6	11.43	54.3	
Children				
no	75	88.6	61.4	0.0002 **
yes	25	11.4	38.6	
Folic acid—before pregnancy				
no	35.7	24.3	47.1	0.0048 **
yes	64.3	75.7	52.9	
Folic acid—during pregnancy				
no	13.6	15.7	11.4	0.4591 **
yes	86.4	84.3	88.6	
Smoking—during pregnancy				
no	98.6	100	97.1	0.1543 **
yes	1.4	0	2.9	
Pre-pregnancy nutrition status on the basis of BMI				
underweight	10	10	10	0.2907 *
norm	75.7	81.4	70	
overweight	11.4	7.2	15.7	
obesity	2.9	1.4	4.3	

*n* number of subjects, * Mann-Whitney U test, ** Chi^2^ test.

**Table 4:** Frequency of consumption of selected caffeinated products, in total and by group.

Product	Total, *n* = 140 X ± SD	Antenatal Classes, *n* = 70 X ± SD	Gynecological Clinic, *n* = 70 X ± SD
ground coffee	1.91 ± 1.56	1.80 ± 1.47	2.03 ± 1.64
instant coffee	2.33 ± 1.75	2.11 ± 1.44	2.54 ± 2.00
black tea (bags) *	4.09 ± 2.40	4.46 ± 2.34	3.71 ± 2.43
black tea (leaf)	1.76 ± 1.43	1.77 ± 1.40	1.74 ± 1.47
green tea (bags)	2.09 ± 1.6	2.09 ± 1.47	2.10 ± 1.74
green tea (leaf)	1.91 ± 1.50	1.97 ± 1.52	1.86 ± 1.49
cola-like drinks	2.04 ± 1.15	1.91 ± 1.11	2.16 ± 1.18
energy drinks	1.10 ± 0.30	1.03 ± 0.17	1.17 ± 0.38
cacao	2.23 ± 1.39	2.24 ± 1.27	2.21 ± 1.5
bitter chocolate	1.96 ± 1.36	1.99 ± 1.36	1.94 ± 1.38
milk chocolate	2.49 ± 1.48	0.44 ± 1.46	2.53 ± 1.50

Answer scale: 3 × day—8, 2 × day—7, 1 × day—6, 5–6 × week—5, 3–4 × week—4, 1–2 × week—3, less than 1 × week—2, never—1; *n*—number of women, X—arithmetic mean, SD—standard deviation; * *p* = 0.0430, Mann-Whitney U test.
